# Case report: Meningoencephalocele and recurrent bacterial meningitis in chronic idiopathic intracranial hypertension

**DOI:** 10.1186/s12883-022-02959-w

**Published:** 2022-11-11

**Authors:** Jinru Zhang, Jianchao Wu, Yue Wu, Yaye Wang, Jingzhe Han

**Affiliations:** 1 Department of Neurology, The Third Hospital of Shijiazhuang, 050000 Shijiazhuang, Hebei China; 2 Department of Emergency, Hengshui People’s Hospital, 053000 Hengshui, Hebei China; 3 Department of Neurology, Xuanwu Hospital, Capital Medical University, 100053 Beijing, China; 4 Department of Neurology, Hengshui People’s Hospital, No. 180 of Renmin East Road, Taocheng District, 053000 Hengshui, Hebei China

**Keywords:** Meningoencephalocele, Recurrent bacterial meningitis, Chronic idiopathic intracranial hypertension

## Abstract

**Background:**

Meningoencephalocele is a rare malformation caused by congenital and acquired lesions. The association between recurrent bacterial meningitis and meningoencephaloceles with cerebrospinal fluid (CSF) leak is reported in the literature. We report a rare case of meningoencephalocele secondary to chronic idiopathic intracranial hypertension as a result of hospitalization repeatedly for meningitis due to the lack of CSF leak.

**Case presentation:**

This study presents a case of a patient with a decade of recurrent meningitis. With clinical symptoms and imaging examination with chronic idiopathic intracranial hypertension, this patient was diagnosed with meningoencephalocele. With the treatment of acetazolamide to decrease CSF product, the patient had no recurrence of meningitis over the 6-months follow-up period.

**Conclusion:**

In patients with recurrent intracranial infections but no history of immunodeficiency, cranial trauma, or neurosurgery, the possibility of meningitis should be considered appropriately, even in the absence of CSF otorrhea or rhinorrhea.

## Background

Meningoencephalocele is a herniation of the intracranial contents such as nerve tissue, cerebrospinal fluid (CSF), and meninges due to congenital or acquired lesions. The etiology of primary meningoencephalocele is unknown, and acquired factors include inflammation, tumors, trauma, and elevated intracranial pressure [1,2]. This disease usually occurs in the medial line, with a few to the side. Depending on the anatomic site of herniation, the disease is divided into 5 types (frontal-ethmoid bone area, sphenoid-orbital area, sphenoid bone-maxillary sinus area, nasopharyngeal area, and ear canal area) [2].

Typical clinical manifestations in patients with meningoencephalocele include headache, seizures, and symptoms of meningitis. Usually, patients experience rhinorrhea and otorrhea of CSF. Moreover, these features depend on the type of the patient’s disease and the location of the brain tissue involved [3,4]. Frequent diagnoses are made based on the presence of meningoencephalocele combined with otorrhea or rhinorrhea of the CSF. However, patients presenting only with recurrent meningitis and lacking CSF leak symptoms are rarely reported and are difficult to diagnose. This study reports a patient with meningoencephalocele secondary to chronic idiopathic intracranial hypertension who presented with recurrent meningitis and lack of CSF leak.

## Case presentation

A 54-year-old woman presented at the neurology department of the Hengshui People’s Hospital with unconsciousness, fever, nausea, and vomiting for one day. In the past ten years, the patient had been hospitalized repeatedly with the same symptoms and complained of suffering from chronic headaches. On admission, her unconsciousness was graded as GCS 7 (2 + 1 + 4), and further examination revealed positive meningeal irritation. Blood tests returned normal results for renal function, liver biochemistry, and white blood cell count. Head computed tomography (CT) found no abnormalities. A lumbar puncture was performed yielding a pressure of 200 mm H_2_O (100–180 mm H_2_O); 374 white blood cells (lymphocytes 20%, neutrophils 80%) (0–10); protein, 324.6 mg/dL (15-45 mg/dL); glucose, 0.91 mmol/L (2.5–4.4 mmol/L) and chloride, 109.5mmol/L (120–130 mmol/L). The CSF cultures was negative, while CSF mNGS did not reveal specific pathogenic microorganism. Patient was started on isoniazid, rifampicin, pyrazinamide, and ceftriaxone, based on a possible diagnosis of tuberculous meningitis and pyogenic meningitis. On the 12th day of admission, the patient’s symptoms gradually resolved, and her vital signs normalized. Typical results included: lumbar puncture pressure,190 mm H_2_O; white blood cells, 20; protein, 80.8 mg/dL; glucose, 2.18 mmol/L and chloride, 119.6 mmol/L.

**Fig. 1:**
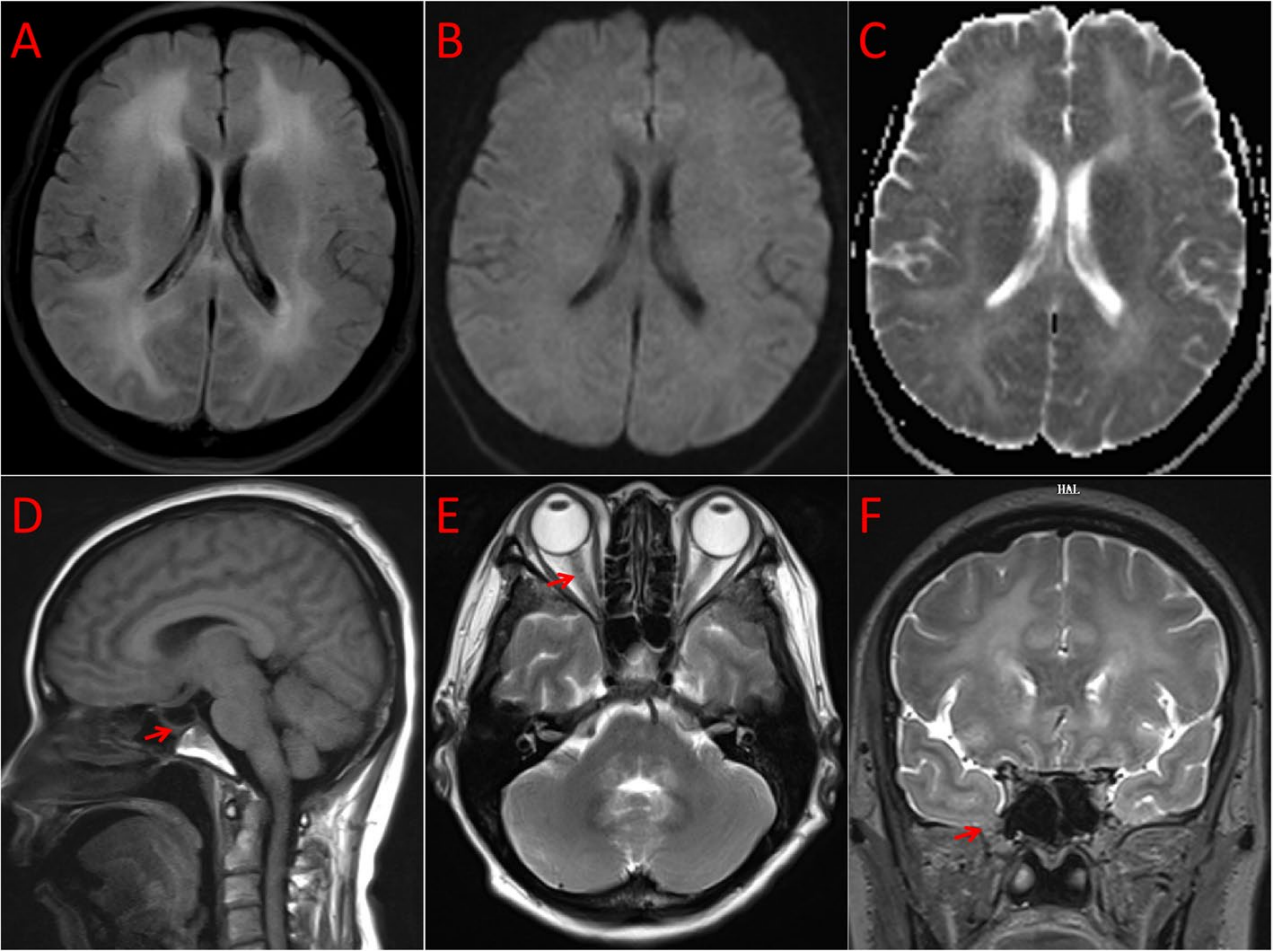
MRI results of the patient. The MRI showed a high FLAIR signal, a standard signal for DWI and ADC in the periventricular white matter (**A**-**C**). Sagittal T1 MRI showed a partially empty sella (**D**, arrowhead). T2 and MRI showed the distention of the optic nerve sheath (**E**, arrowhead). Coronal T2 and MRI showed right meningoencephaloceles with herniating of the temporal lobe and surrounding CSF on the right (**F**, arrowhead)

However, the patient developed various symptoms 17 days after admission, including persistent fever, headache, nausea, and vomiting. Lumbar puncture revealed an opening pressure greater than 300 mm H_2_O, with the CSF adopting a rice soup-like appearance. The WBC count was 2911; protein,178.2 mg/dL; glucose, 1.82 mmol/L and chloride, 118.6 mmol/L. The CSF cultures showed hemolytic streptococcus. Subsequently, we used meropenem and norvancomycin to treat patient with possible bacterial meningitis. In the meantime, we embarked on searching for the cause of recurrent meningitis. Magnetic resonance imaging (MRI) scan of the brain showed multiple symmetrical abnormal signals around the white matter of the fourth ventricle, brainstem, bilateral posterior limbs of the internal capsule, and both cerebral hemispheres. In addition, a partially empty sella, tortuous optic nerve sheaths, flattening of the optic papilla, and narrowing of the ventricle was evident. The previous findings were consistent with chronic idiopathic intracranial hypertension (IIH). Coronal T2 MRI revealed right meningoencephaloceles with herniation of the temporal lobe and surrounding CSF (Fig. 1). Then, digital subtraction angiography (DSA) of the brain demonstrated dysplasia of the left transverse sinus and slow venous return with no definite vascular malformation. Eventually, bacterial meningitis, IIH, and meningoencephalocele were diagnosed. However, the patient did not consent to surgical repair and CSF shunting procedure.

Consequently, she was treated with acetazolamide. On day 28 of admission, the lumbar puncture had an opening pressure of 180 mm H_2_O and 10 white blood cells; protein, 60.8 mg/dL; the glucose (3.82 mmol/L) and chloride (121.6 mmol/L) were within normal values. The patient continued taking regular oral acetazolamide without reporting recurrent meningitis six months after being discharged from the hospital.

## Discussion and conclusions

Meningoencephalocele is primarily a congenital condition, but trauma, inflammation, postoperative complications, and neoplasms have also been implicated in its pathogenesis. IIH has also been observed to cause meningoencephalocele and spontaneous CSF leakage [5]. An imbalance causes IIH in the secretion and absorption of CSF due to a variety of possibly unknown causes. On neuroimaging, an empty sella turcica, widening of the optic nerve sheath, and fissure-like changes in the anterior horn of the lateral ventricle are typical features of IIH [6]. The MRI scan of the patient’s brain at admission was consistent with the typical neuroimaging features of IIH. However, we ruled out the diagnosis of IIH at the outset, based on the diagnostic criteria for the IIH CSF component and the patient’s clinical features. We shifted our diagnostic focus to finding the etiology of chronic infections and imaging changes in specific infections. This must have played a significant role in the delayed diagnosis and treatment of the patient. Increased CSF pressure may also be associated with acute meningitis, but the headache and MRI findings suggest the patient had prolonged intracranial hypertension, which further supports the diagnosis of IIH. The differential diagnosis of IIH includes cerebral venous sinus thrombosis and dural arteriovenous fistulas. The patient’s brain’s DSA did not find any features related to the differential mentioned above diagnoses; therefore, they were excluded. It is generally believed that intracranial venous sinus stenosis is one of the causes of IIH. In several studies, transverse sinus stenosis was found in 90% of IIH patients [7]. The DSA of the patient’s brain found dysplasia of the left transverse sinus and slow venous return, which may cause increased intracranial pressure. The meningoencephalocele, secondary to IIH, may be due to the pressure effects of the brain tissue and CSF, causing enlargement of the original defect or fragile bony structure and may lead to thinning bony structures.

The location of the temporal meningoencephalocele (anteromedial, anterior inferior, posterior inferior, anterior, and lateral) provides the basis for its classification and the anticipated clinical manifestations. Temporal anteromedial meningoencephalocele refers to the herniation of the anterior medial wall of the middle cranial fossa into the sphenoid sinus, accompanied by CSF rhinorrhea. The condition can be detected by conducting CT and MRI scans of the brain. In this case report, an abnormal signal in the medially protruding right temporal lobe was identified using a 0.7 mm, 0-space thin-layer 3D-T2-space sequence, which led to the identification of the patient as having a meningeal brain meningoencephalocele. Patients with temporal anteromedial meningoencephalocele are susceptible to bacterial meningitis. In such a case, bacteria can directly infect meninges through the cranial pathway, such as the site of cranial incision, middle ear, sinus inflammation, skull fracture, or brain trauma, leading to bacterial meningitis. The presence of the right meningocele in the patient’s nasal cavity makes it easier for bacteria to enter the meninges and ventricles through the intracranial and nasal passages. It triggers inflammation, resulting in complex symptoms including headache, vomiting, fever, features of meningeal irritation, and neurolocalization signs of acute bacterial meningitis.

The correct diagnosis for this patient was only achieved after ten years because of the atypical symptoms of bacterial meningitis. Previously, the patient had been hospitalized repeatedly for viral encephalitis, tuberculous and suppurative meningitis. The patient’s initial diagnosis on admission was probable tuberculous meningitis. However, a significant improvement in the patient’s condition occurred, together with a negative pathogenic microorganisms screening result and a recurrence of meningitis, confirming the diagnosis of bacterial meningitis. It remains to be established what causes the atypical clinical symptoms and signs. Probable contributors to this observation are the self-limiting nature of certain infections, the protective effect of the meninges that provide a barrier to infection, and the unique patient-dependent resistance to infections.

To avoid recurrence, the standard of care for IIH includes surgical correction of the meningoencephalocele and subsequent medical treatment [5]. However, the patient did not consent to surgery and opted for therapeutic management to decrease CSF production. At six months of follow-up, the patient had no recurrence of meningitis. The long-term success of this treatment approach requires further observation with longer follow-ups. The periventricular white matter edema observed during the examination of the patients is also related to impaired venous return related to elevated cranial pressure. This will require further monitoring using neuroimaging during follow-up.

In conclusion, this is a rare case of meningoencephalocele associated with recurrent meningitis in patients without a CSF leak. Chronic idiopathic intracranial hypertension might also cause meningoencephalocele. The coronal view MRI brain scan of a patient with recurrent bacterial meningitis is required to examine the presence of meningoencephalocele. This should be considered even in the absence of CSF otorrhea or rhinorrhea.

## Data Availability

The datasets used or analyzed during the current case reports are available from the corresponding author on reasonable request.

## Abbreviations

CSF: Cerebrospinal fluid
CT: Computed tomography
MRI: Magnetic resonance imaging
IIH: Idiopathic intracranial hypertension
DSA: Digital subtraction angiography
